# Postmenopausal Hyperandrogenism Associated With Synchronous Ovarian Brenner Tumor, Bilateral Leydig Cell Tumor, and Adrenal Mass

**DOI:** 10.7759/cureus.55334

**Published:** 2024-03-01

**Authors:** Michael Salim, Sandhyarani Dasaraju, Britt Erickson, Mahmoud Khalifa, Lynn A Burmeister

**Affiliations:** 1 Endocrinology, Diabetes, and Metabolism, University of Minnesota School of Medicine, Minneapolis, USA; 2 Laboratory Medicine and Pathology, University of Minnesota School of Medicine, Minneapolis, USA; 3 Gynecologic Oncology, University of Minnesota School of Medicine, Minneapolis, USA

**Keywords:** virilizing adrenal tumor, brenner tumor of the ovary, adrenal mass, venous sampling, testosterone in women, leydig cells, ovarian sex cord-stromal tumor, ovarian tumor, hyperandrogenism, postmenopausal female

## Abstract

Hyperandrogenism in postmenopausal females may arise from either ovarian or adrenal sources and can pose a challenging diagnostic dilemma. We present the case of a 66-year-old female with postmenopausal hyperandrogenism with virilization, adrenal incidentaloma, and concurrent finding of two extremely rare ovarian tumors, including bilateral Leydig cell tumor and Brenner tumor. Laboratory tests showed elevated testosterone and androstenedione and normal dehydroepiandrosterone sulfate (DHEAS). Response to 1 mg overnight dexamethasone suppression test demonstrated persistently elevated testosterone and incomplete suppression of androstenedione. Computed tomography (CT) scan showed a left adrenal nodule and an unremarkable appearance of the ovaries. The pelvic ultrasound did not show an ovarian tumor on the right ovary, and the left ovary was not seen. Adrenal and ovarian vein sampling suggested the ovaries as the source of the testosterone. Given the ovarian vein sampling results, a multidisciplinary discussion between endocrinology and gynecologic oncology concluded that bilateral salpingo-oophorectomy (BSO) was the next best step for diagnosis and management. Laparoscopic BSO was performed. Histopathology showed bilateral Leydig cell tumors and a left ovarian Brenner tumor. At one-year postoperative follow-up, alopecia improved, and testosterone level normalized. This case highlights the importance of diagnostic pathways and interdisciplinary collaboration in managing rare clinical scenarios of hyperandrogenism in postmenopausal females. As in our case, surgeons may be hesitant to remove normal-appearing ovaries. While the three presented tumor types in this case arise from distinct tissues and exhibit different histological characteristics, the presence of such a unique triad prompts consideration of potential unifying pathogenic mechanisms.

## Introduction

New-onset hyperandrogenism is rare after menopause [1-3]. Postmenopausal hyperandrogenism may arise from either ovarian or adrenal sources [2,3]. Androgen-secreting tumors are the least common cause of clinical hyperandrogenism, found in only 0.2% of cases [1].

We present the case of a 66-year-old female with postmenopausal hyperandrogenism with virilization, adrenal incidentaloma, and concurrent finding of two extremely rare ovarian tumors, including bilateral Leydig cell tumor and Brenner tumor.

## Case presentation

A 66-year-old multigravida, postmenopausal female presented with a three-month history of worsening alopecia. Her past medical history included diabetes mellitus type 2, hypertension, obstructive sleep apnea syndrome, breast cancer, osteoporosis, gastroesophageal reflux, and coronary artery disease. Menarche was at age 12. There was no history of infertility. Menopause was in her 40s. Breast cancer diagnosis was at age 55. The tumor was an estrogen receptor-positive left-sided invasive ductal carcinoma, pT2, pN0, M0 stage IIA. Treatment was left mastectomy, chemotherapy, and aromatase inhibitor for five years. She had been shaving her upper lip and chin hair every three days for the past 10 years. Scalp hair loss, with loss of the bangs, had accelerated in the last three months. The physical examination was significant for body mass index of 34, deep voice, and clitoromegaly. Laboratory tests showed testosterone of 160 ng/dL (reference range: 8-60 ng/dL), free testosterone of 3.37 ng/dL (reference range: 0.06-0.38 ng/dL), androstenedione of 219.6 ng/dL (reference range: 13-82), sex hormone binding globulin of 31 nmol/L (reference range: 30-135 nmol/L), dehydroepiandrosterone sulfate (DHEAS) of 86 ug/dL (reference range: 13-130 ug/dL), 17-hydroxy progesterone of 96 ng/dL (reference range: <51 ng/dL), inhibin A of 1.1 pg/mL (reference range: <6.9 pg/mL), inhibin B of <10 pg/mL (reference range: <16 pg/mL), adrenocorticotropic hormone of 38 pg/mL (reference range: <47 pg/mL), and cortisol of 12.2 ug/dL (reference range: 4-22 ug/dL). Overnight dexamethasone suppression test (1 mg) demonstrated persistently elevated testosterone (160 ng/dL) and incomplete suppression of androstenedione (82.7 ng/dL) and cortisol (2.4 ug/dL).

**Figure 1:**
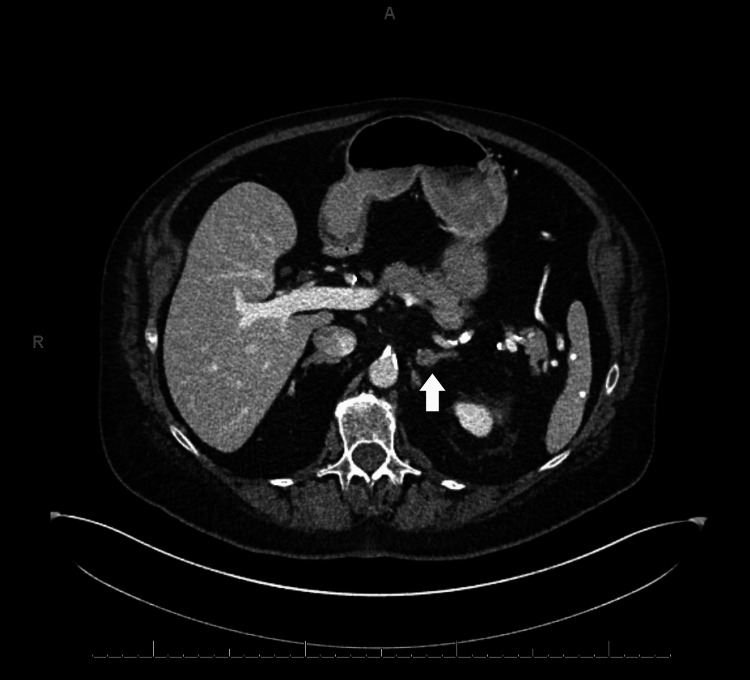
CT scan with contrast of the abdomen showed a 12 mm left adrenal nodule CT: computed tomography

**Figure 2:**
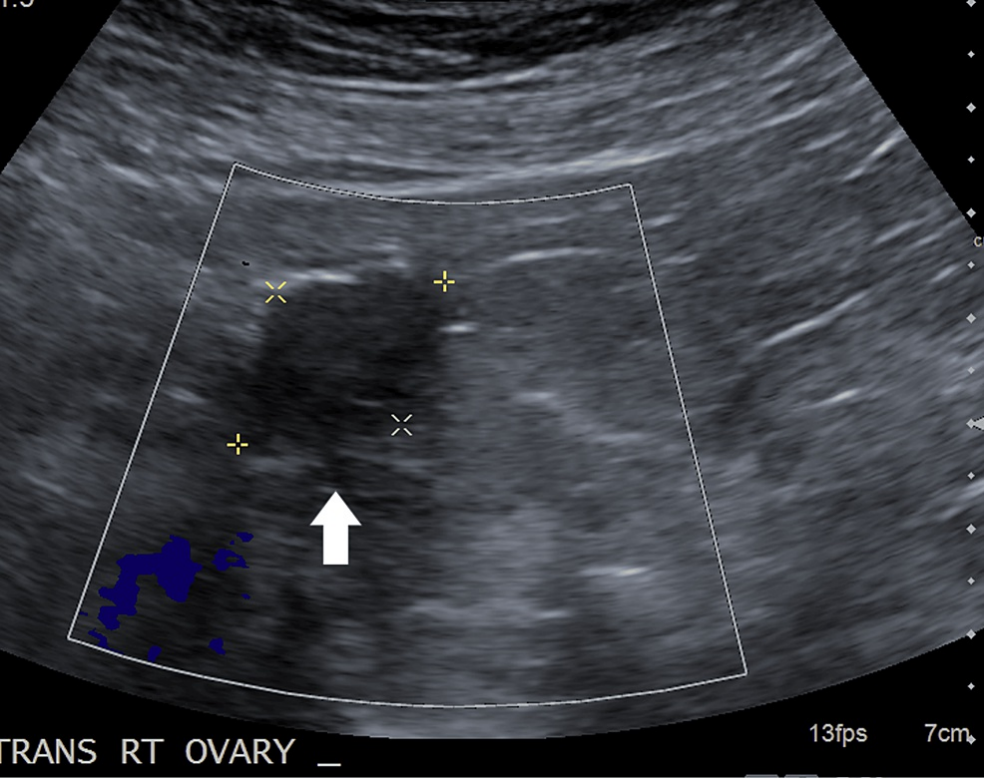
Ultrasound of the right ovary (arrow) A transvaginal ultrasound of the right ovary did not show a tumor. The ovary has a normal size measuring 2.5 × 2.5 × 1.7 cm. The left ovary was not visualized.

Computed tomography (CT) scan with contrast showed a left adrenal nodule (12 mm, stable compared to imaging two years prior when the nodule measured 5 Hounsfield units on a non-contrast examination) (Figure 1) and an unremarkable appearance of the ovaries. The pelvic ultrasound did not show an ovarian tumor on the right, and the left ovary was not seen (Figure 2).

Adrenal and ovarian vein sampling, which was performed by an experienced interventional radiologist, suggested the bilateral ovaries as the source of the testosterone (Table 1).

**Table 1:** Adrenal and ovarian vein sampling Ovarian and adrenal vein sampling data: 250 mcg cosyntropin infusion was given during the procedure, resulting in the expected increase in cortisol, androstenedione, and aldosterone. Testosterone output from the mean ovaries exceeded that of the IVC by 4,019 ng/dL and the mean adrenal glands by 267 ng/dL. The ovarian testosterone-to-IVC fold ratio was as follows: left ovary, 21 and right ovary, 14. The left-to-right ovarian gradient for testosterone was 1.4, and for androstenedione, 1.6. Normal reference ranges are as follows: androstenedione postmenopausal, 0.13-0.82 ng/dmL; cortisol, 4-22 mcg/dL at 8 AM; testosterone, 8-60 ng/dL; and aldosterone, 0-31 ng/dL. Ovarian venous sampling access was obtained through ultrasound-guided right internal jugular vein puncture, microguidewire advancement, and placement of a 12-French vascular sheath within the IVC. Catheterization and sampling of the right ovarian vein were followed by sampling of the left ovarian vein. Ultrasound-guided right or left common femoral vein puncture was followed by the placement of additional 7-French sheaths in the IVC. Angiography confirmed positioning from the right common femoral vein to the left renal vein, left adrenal-phrenic trunk, and sampling of the left adrenal vein through a Simmon 2 catheter and from the left common femoral vein to the right adrenal vein sampling through a Kumpe catheter. Blood samples were obtained from the adrenal veins, as well as the above and below inferior vena cava regions for purposes of adrenal vein sampling. IVC: inferior vena cava

	IVC below	IVC above	Right ovary	Left ovary	Left-to-right ovary ratio	Right adrenal	Left adrenal
Androstenedione (ng/mL)	3.7; 3.9	5.3; 4.07	12.97	21.23	1.64	>40	>40
Cortisol (ug/dL)	54.5; 42.6	49.7; 52.8	-	-	-	1,336; 1,459	399; 462.8
Testosterone (ng/dL)	161; 171	500; 181	3,725	5,326	1.43	588; 694	347; 449
Aldosterone (ng/dL)	19.4; 18.4	22.8; 19.2	-	-	-	451; 555	999; 106

**Figure 3:**
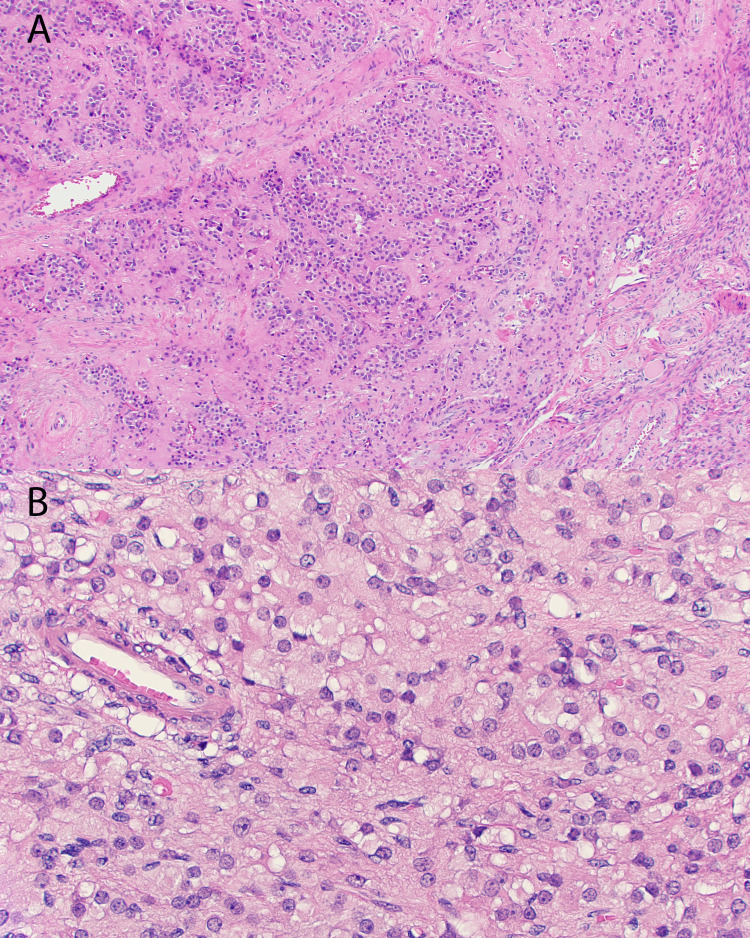
Leydig cell tumor Leydig cell tumor composed of sheets of uniform cells with abundant vacuolated eosinophilic cytoplasm and prominent nucleoli (200× (A) and 400× (B)).

**Figure 4:**
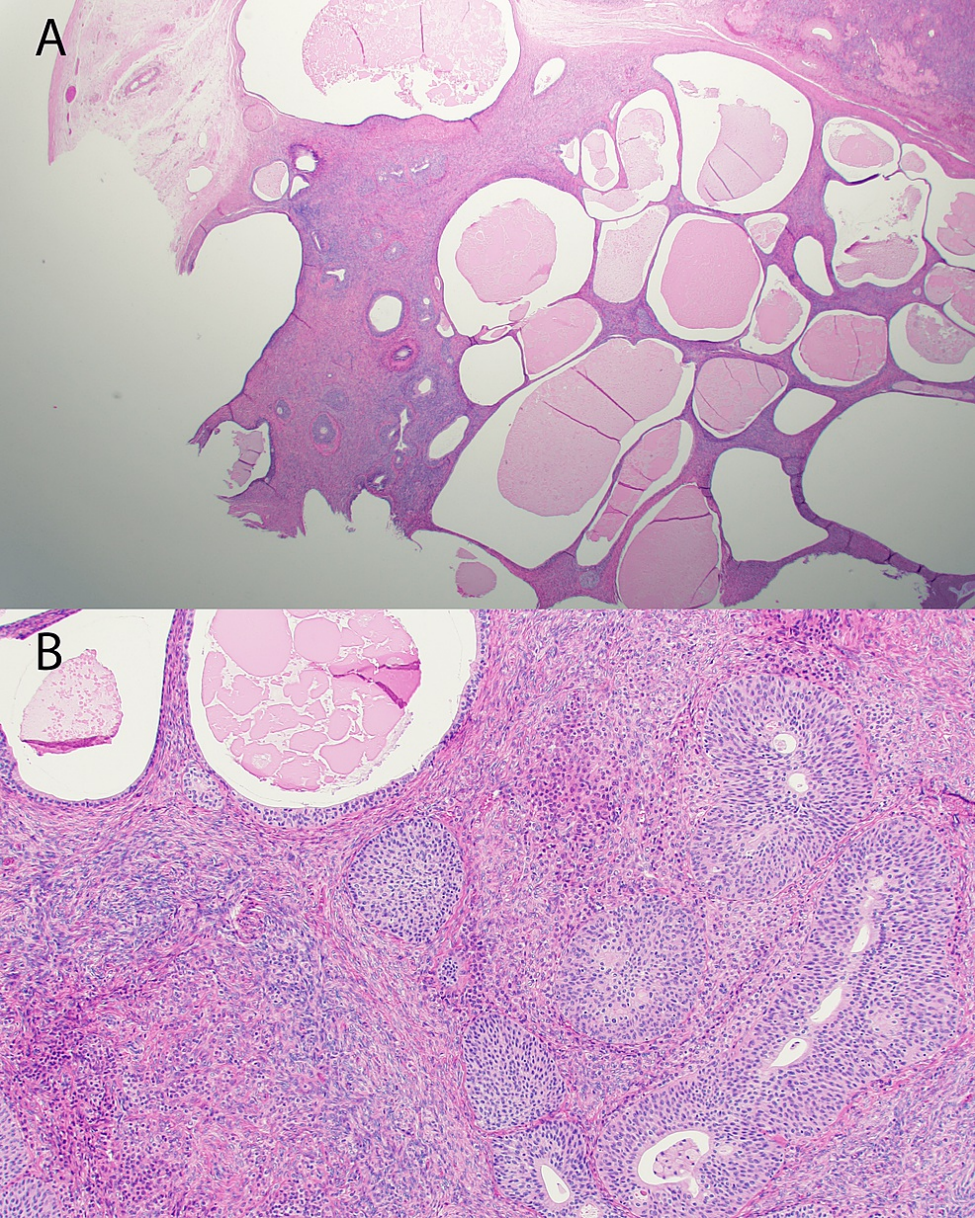
Brenner tumor Brenner tumor composed of nests of bland transitional epithelium with extensive mucinous metaplasia (100× (A) and 200× (B)).

A multidisciplinary discussion between endocrinology and gynecologic oncology was followed by laparoscopic bilateral salpingo-oophorectomy (BSO). Intraoperatively, the ovaries appeared grossly normal with dilated ovarian veins. Histopathology showed bilateral Leydig cell tumors (left: 1.5 cm, right: 2 cm) and a 0.7 cm left ovarian Brenner tumor (Figure 3 and Figure 4).

At one-year postoperative follow-up, alopecia had resolved, and total (23 ng/dL) and free testosterone (0.47 ng/dL) had normalized. Androstenedione (102.3 ng/dL) was lower than preoperatively but higher than the reference range.

## Discussion

We report a challenging diagnostic case of postmenopausal virilization leading to the finding of concurrent bilateral ovarian Leydig cell tumor, Brenner tumor, and adrenal incidentaloma. To our knowledge, this is the first case of synchronous Brenner tumor and bilateral Leydig cell tumor and the 12th English language report of bilateral Leydig cell tumor. Worsening alopecia and virilization led to the discovery of high testosterone and androstenedione along with an adrenal nodule in a 66-year-old female. Transvaginal ultrasound and pelvic CT found normal-appearing ovaries. Overnight dexamethasone suppression test partially suppressed cortisol and androstenedione, but testosterone did not change. Adrenal and ovarian vein sampling demonstrated an ovarian-to-peripheral testosterone gradient > 77 ng/dL and a right-to-left ovarian ratio < 1.44, supporting a bilateral ovarian source [4]. Bilateral oophorectomy histopathology revealed a bilateral Leydig cell tumor (left, 1.5 cm; right, 2 cm), with a 0.7 cm Brenner tumor. The testosterone level normalized, and alopecia resolved on follow-up.

New onset or significant progression of clinical hyperandrogenism or virilization is extremely rare in postmenopausal females, where it is usually due to either ovarian hyperthecosis or an androgen-secreting tumor [2,3]. Distinguishing the source (ovarian hyperthecosis versus tumor and ovarian versus adrenal) becomes challenging because both ovarian and adrenal tumors can overproduce the same hormones, including testosterone, DHEAS, and androstenedione [5-7]. Testosterone above 64 ng/dL defines pathologic postmenopausal hyperandrogenism [2]. Algorithms based on hormone levels, most commonly testosterone and DHEAS, imaging findings, or response to dexamethasone or gonadotropin-releasing hormone (GnRH) have been reported [3,6,8,9]. Tumorous testosterone levels are typically over 144 ng/dL (5 nM) to 150 ng/dL, as seen in our patient [2,3,8]. While high DHEAS is more typical of adrenal tumors, ovarian tumors can also present with high DHEAS levels, and 18% of androgen-secreting adrenocortical tumors had normal DHEAS levels [5,6,10]. Incomplete dexamethasone-induced suppression of cortisol and androstenedione and no change in testosterone were suggestive of ovarian source in our patient. Given the presence of adrenal mass and normal-appearing ovaries, ovarian and adrenal vein sampling sought to discriminate between adrenal or ovarian location and its laterality [4,6,7,9]. The mean ovarian-to-vena cava testosterone effluent was much greater than that of the adrenal (4,019 ovarian and 267 adrenal), supporting an ovarian source. The left-to-right ovarian effluent ratio was consistent with other cases of bilateral ovarian disease. However, given a lack of guidelines for the performance and interpretation of venous sampling results, the gynecologic surgeon did hesitate before performing a bilateral oophorectomy. Histopathology, the gold standard, proved the presence of bilateral Leydig cell tumors, measuring 1.5 and 2 cm, with a 0.7 cm left Brenner tumor.

Most tumors of the adrenal or ovary do not produce excess androgen. Androgen-producing tumors accounted for only 5.3% (10/190) of adrenal tumors reported from a surgical series [10]. Likewise, it is estimated that only 1% of ovarian tumors secrete androgens [6]. Androgen may be produced by ovarian tumor subtypes classified as sex cord-stromal tumors (which includes pure Leydig cell tumors) or germ cell tumors and even more rarely with Brenner tumors [6].

Leydig cell tumor, formerly called hilus cell tumor, is a subtype of the WHO ovarian neoplasm sex-cord stromal tumor category [11]. Leydig cell tumor cells are morphologically identical to testicular Leydig cells, including the potential presence of cytoplasmic inclusions known as Reinke crystalloids [12]. These rare tumors typically present in postmenopausal females as a small unilateral lesion, difficult to identify on imaging [6,13]. In a tertiary center surgical series of 957 ovarian neoplasms, only 5.6% were sex-cord stromal tumors, with no pure Leydig cell tumors, speaking to their rarity [14]. Bilateral Leydig cell tumors have been reported only 12 times in the English language literature, including this case (Table 2).

**Table 2:** Case reports with bilateral Leydig cell tumor English language case reports A: androstenedione, DHEA: dehydroepiandrosterone, DHEAS: dehydroepiandrosterone sulfate, H: hirsutism, A: alopecia, C: clitoromegaly or prominent clitoris, Ac: acne, V: voice deepening, L: increased libido, PB: postmenopausal bleeding, NR: not reported, BL: bilateral, MNG: multinodular goiter, PTC: papillary thyroid carcinoma, USp: ultrasound of the pelvis, USa: ultrasound of the abdomen, CTab: computed tomography of the abdomen, CTpel: computed tomography of the pelvis, MRI: magnetic resonance imaging, PET: positron emission tomography

First author, year	Age at diagnosis	Clinical presentation	Testosterone (ng/dL) (reference range)	A (ng/dL) (reference range)	DHEA (ug/dL)	DHEAS (reference range)	Leydig cell tumor size (maximum dimension) (mm)	Other endocrine/ovarian tumors	Seen on imaging	Imaging type	Ovarian and adrenal vein sampling
Sternberg, 1949 [12]	86	H, A, C, V	NR	NR	NR	NR	10, 12	No	No	X-ray	NR
Baramki, 1983 [15]	51	H, A, C	362 (36-62)	165 (122-240)	NR	NR	7, 6	Prior hyperthyroidism	No	USp, CTab	NR
Böhm, 1991 [16]	58	H, A, C	176 (<69)	NR	NR	NR	NR	No	No	USp, CTab	NR
Duun, 1994 [17]	72	H, A	"Elevated," NR	NR	NR	NR	9	Prior hyperthyroidism	No	USp, CTab	NR
Kaltsas, 2003 [7]	62	PB	173	45 nmol/L	NR	2.1 nmol/L	"Small" and "multiple"	Left adrenal nodule (2 cm)	R ovary bulky	USp, CTab	Yes
Sanz, 2007 [18]	77	H, A	530 (14-76)	2.9 pg/L (0.4-2.7 pg/L)	NR	NR	15, 3	No	Left ovary lesion (12 mm)	USp, CTab	NR
Marcelino, 2010 [19]	67	H, A	662 (<62)	2 (0.3-3.1)	NR	4.1 ng/dL (0.8-10.5 ng/dL)	10	No	No	USp, CTab, CTpel	NR
Andola, 2019 [20]	65	H, A, C	536 (6-82)	NR	4.7 ng/mL (2-6 ng/mL)	NR	20	No	Right ovary isoechoic lesion	USp, CTab, CTpel	NR
Langevin, 2020 [21]	64	H, A, C, L, PB	234 (8-60)	385 (30-200)	NR	110 mcg/dL (9.7-159 mcg/dL)	NR	MNG	No	USp, CTab, CTpel	NR
Hussain, 2021 [22]	64	H, A, C	242	306 (10-93)	NR	150 mcg/dL	10	Lipid-rich adrenal adenoma (8 mm)	No	USp, CTab, CTpel, MRI, PET scan	NR
Shakir, 2021 [23]	61	H, A, C, Ac, V, L	803 (3-41)	28 (31-701)	512 (31-701)	99.9 mcg/dL (19-220 mcg/dL)	NR	PTC	Homogenous ovarian enhancement BL	USp, USa, CTab; MRI	NR
Salim, present case	66	H, A	160 (8-60)	219.6 (13-82)	N/A	86 (13-130)	15, 20	Left adrenal nodule (12 mm)	No	USp, CTab, CTpel	Yes

All were in postmenopausal females. Where measured, all had high testosterone ≥169 ng/dL. Five of the 12 also had high androstenedione. Three showed unilateral lesions on ovarian ultrasound, and one case showed bilateral ovarian lesions on magnetic resonance imaging (MRI) [7,18,20]. It is remarkable that three of the 12 cases of bilateral Leydig cell tumor, including the present case, also had an adrenal mass [7,22]. Only the case by Kaltsas et al. underwent venous sampling, but the procedure was technically unsuccessful [7].

Brenner tumor is an even rare subtype of the WHO epithelial tumor classification category, accounting for only 17/957 cases (1.77% overall) from a tertiary center surgical series [11,14]. There are only a few reports of Brenner tumor with hyperandrogenism [24]. Aside from this case, there are no other reports of bilateral Leydig cell tumor associated with Brenner tumor. Brenner tumors express androgen receptors and the aldo-keto reductase AKR1C3, a key enzyme in androgen production [24]. As such, it could be hypothesized that Brenner tumor might be stimulated by Leydig cell tumor androgen production. Our patient's Brenner tumor was located on the left ovary, possibly explaining the higher testosterone effluent on the left than on the right ovary.

## Conclusions

In conclusion, the presented case describes the intriguing clinical scenario of postmenopausal virilization due to the synchronous presence of two rare ovarian tumors, both capable of causing hyperandrogenism, including bilateral ovarian Leydig cell tumor, Brenner tumor, and adrenal incidentaloma. Despite normal-appearing ovaries on imaging studies, BSO was necessary for diagnosis and management. As in our case, surgeons may be hesitant to remove normal-appearing ovaries, and therefore, this case highlights the importance of diagnostic pathways and interdisciplinary collaboration. It is unknown if the 25% rate of bilateral Leydig cell tumor and coexistent adrenal mass may indicate a common pathogenesis. While the three presented tumor types in this case arise from distinct tissues and exhibit different histological characteristics, the presence of such a unique triad prompts consideration of potential unifying pathogenic mechanisms. This case offers novel information for future physicians who encounter these rare clinical scenarios.
